# Endoscopic Management of Bouveret Syndrome Complicated by Jejunal Perforation

**DOI:** 10.14309/crj.0000000000002372

**Published:** 2026-10-21

**Authors:** Meghana N. Vankina, Jayaprakash Sreenarasimhaiah

**Affiliations:** 1 Tulane University School of Medicine, New Orleans, LA; 2 Division of Gastroenterology, Baylor Scott & White, Irving, TX

**Keywords:** Bouveret syndrome, gastric outlet obstruction, endoscopic holmium laser lithotripsy, cholecystoduodenal fistula, jejunal perforation

## Abstract

Bouveret syndrome is a rare cause of gastric outlet obstruction due to gallstone migration through a bilioenteric fistula. We present a 76-year-old woman with multiple comorbidities who developed Bouveret syndrome and was treated with endoscopic holmium laser lithotripsy. Although the obstructing stone was successfully fragmented and removed, the patient later developed a jejunal perforation. She was ultimately transitioned to hospice care. To our knowledge, jejunal perforation after endoscopic laser lithotripsy for Bouveret syndrome has not been previously reported. This case highlights a rare but serious complication of minimally invasive endoscopic management, particularly in high-risk patients with large stones.

## INTRODUCTION

Bouveret syndrome is a rare form of gastric outlet obstruction that is caused by the impaction of a gallstone in the duodenum or stomach after migrating through a bilioenteric fistula.^1,2^ In gallstone ileus, the gallstone most commonly lodges at the ileocecal valve, whereas in Bouveret syndrome the gallstone becomes lodged more proximally. Bouveret syndrome accounts for less than 3% of cases of gallstone ileus and typically presents in elderly women with an average age of 70 years who have multiple comorbidities.^1,3^ The clinical presentation is often nonspecific, but symptoms can include nausea, vomiting, abdominal pain, abdominal distension, signs of dehydration, and recent weight loss.^3^ Risk factors and causes include longstanding cholelithiasis, chronic cholecystitis, and formation of a bilioenteric fistula, of which cholecystoduodenal fistula is most common.

Management is challenging, particularly in high-risk surgical candidates. Surgery has traditionally been the standard of care, but endoscopic techniques are increasingly being used as it can be more minimally invasive. We present a case of Bouveret syndrome managed with endoscopic holmium laser lithotripsy complicated by jejunal perforation.

## CASE REPORT

The patient is a 76-year-old woman with a medical history of known gallstones and chronic cholecystitis, which was being managed with a cholecystostomy tube for over 18 months, but was removed 2 months prior to admission. Other comorbidities included coronary artery disease, heart failure with preserved ejection fraction, chronic kidney disease, and chronic obstructive pulmonary disease. On examination, she appeared anxious and tearful, with multiple episodes of emesis. She presented to the emergency department with midsternal chest pain radiating to her back, along with nausea, vomiting, and abdominal distension; cardiac etiology was ruled out with a normal electrocardiogram, negative serial troponins, and an unremarkable chest X-ray.

Laboratory results were unremarkable apart from a mildly elevated white blood cell count (12.0 × 10^9^/L), mild hypokalemia, and slightly elevated creatinine of 1.3–1.4 mg/dL (baseline 1.2 mg/dL). Given her abdominal distension, computed tomography (CT) of the abdomen and pelvis was ordered. which revealed a large 4 cm calcified gallstone within the distal stomach (Figure 1), pneumobilia, and gastric distention, with no evidence of small bowel pathology or distension. These findings were consistent with Bouveret syndrome. Given her cardiac history and comorbidities, she was deemed to be a poor surgical candidate and was initially managed with nasogastric decompression. After multidisciplinary risk discussion, she underwent upper endoscopy, which revealed a fistulous tract in the posterior duodenal bulb entering the gallbladder, confirming a cholecystoduodenal fistula (Figure 1) rather than the cholecystogastric fistula suggested by the stone's apparent location on CT, and a large 4 cm round gallstone impacted at the duodenal bulb causing gastric outlet obstruction. Endoscopic holmium laser lithotripsy was then performed using an Olympus PCF-190 pediatric colonoscope at a power setting of 12 W, 1.0 J per pulse, and a frequency of 10 Hz, with a total of 6,690 laser pulses applied (Figure 1). The laser fragmented the stones into small pieces less than 1 cm and mechanical extraction with a Roth net, rat-tooth forceps, and snare was then used to remove the accessible fragments. The remaining pieces were small enough to be flushed downstream. Normal saline (0.9% NaCl) was used for fluid immersion during laser therapy. The total procedure duration was approximately 128 min, reflecting the complexity of stone fragmentation and extraction.

**Figure 1.**
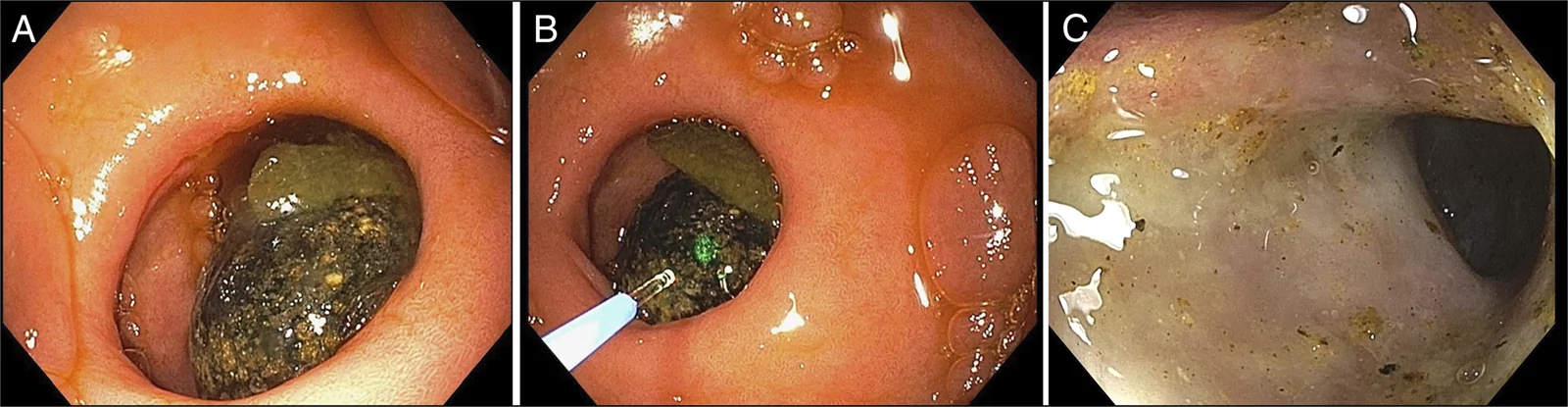
Endoscopic findings in Bouveret syndrome. (A) Large impacted gallstone in the duodenal bulb causing gastric outlet obstruction. (B) Holmium laser lithotripsy applied to fragment the gallstone. (C) Cholecystoduodenal fistula visualized in the posterior duodenal bulb, seen as an opening in the posterior wall of the duodenal bulb with the lumen of the duodenum in the foreground.

Twelve hours postprocedure, the patient developed new-onset abdominal pain. Repeat CT imaging with oral contrast revealed extravasation of contrast into the central small bowel mesentery, with mild thickening of the jejunum and no significant surrounding inflammatory changes, consistent with a jejunal perforation. No residual calcified stone fragments were identified on imaging. Surgical exploration and repair were initially recommended as the gold-standard treatment, but anesthesia deemed the patient a prohibitive risk for general anesthesia given her cardiopulmonary comorbidities. In keeping with her previously expressed wishes to avoid further invasive procedures, a multidisciplinary discussion led to a comfort-based approach that aligned with the patient's wishes. She remained hospitalized for symptom management and was later discharged with home hospice services. An autopsy was not performed.

## DISCUSSION

Bouveret syndrome is an uncommon cause of gastric outlet obstruction in elderly patients with multiple comorbidities. The management remains challenging due to the lack of standardized guidelines and the need to individualize treatment based on patient risk factors and stone characteristics.

In this case, a 76-year-old woman with chronic cholecystitis presented with nausea, vomiting, abdominal distension, and chest pain. A broad differential was considered, but cardiac workup was negative. Bouveret syndrome was ultimately diagnosed based on imaging findings. Abdominal/pelvic CT is the most sensitive modality and typically shows a gallstone within the duodenum or pylorus, pneumobilia, and gastric distention.^4^

Surgery, via gastrotomy or duodenotomy, has traditionally been the primary treatment approach for Bouveret syndrome.^1,2^ However, in recent years, advancements in minimally invasive endoscopic approaches are being favored, especially in elderly or high-risk patients due to lower morbidity rates.^5,6^ Endoscopic management options include mechanical extraction and various lithotripsy techniques.^5–7^ However, success can be limited in large or impacted stones, and fragmentation carries the risk of distal migration. Holmium laser lithotripsy allows for precise stone fragmentation and is effective for large stones with minimal injury to surrounding tissue.^5,6^

The mechanism underlying this patient's jejunal perforation is most likely passage of a sharp-edged stone fragment through the bowel lumen, producing direct mechanical injury distal to the lithotripsy site. Direct thermal injury from the laser is unlikely given its highly localized effect at the point of stone contact, far proximal to the jejunum. In addition, the absence of residual calcified fragments on CT does not completely exclude alternative mechanisms, including transient pressure-related ischemia or localized mural injury during fragment passage, highlighting the inherent uncertainty in interpreting a single case. Notably, there was no evidence of small bowel pathology or distension on the CT preceding the procedure, supporting that the perforation occurred as a consequence of lithotripsy rather than a preexisting process. Other potential contributing factors, such as excessive insufflation during a prolonged endoscopy, are possible but seem less likely given the absence of any preprocedural bowel abnormality and the temporal relationship to stone fragment passage.

While jejunal perforation following endoscopic lithotripsy has not been widely reported, this case highlights a potential risk of distal bowel injury from sharp stone fragments. The most common complications reported after endoscopic management of Bouveret syndrome are distal gallstone ileus due to the migration of stone fragments or mucosal injury at the site of the impaction.^8,9^ Although cases have demonstrated the efficacy and safety of holmium laser lithotripsy in relieving obstructing gallstones, this case raises awareness of a rare but serious postlaser lithotripsy complication that can occur and reinforces the need for careful postprocedural monitoring. Stones larger than 2.5–3 cm may have a greater risk of downstream obstruction or trauma during migration, and fragment size and retrieval strategy should be considered in procedural planning. Frank bowel perforation following endoscopic lithotripsy for Bouveret syndrome is rare. On review of the literature, we identified 1 report of duodenal perforation after endoscopic lithotripsy that was managed conservatively.^10^ To our knowledge, jejunal perforation following endoscopic laser lithotripsy for Bouveret syndrome has not been previously reported, distinguishing this case from the more commonly described complication of distal gallstone obstruction. Several procedural modifications may help mitigate the risk of fragment-related complications. Periodic retrieval of stone fragments throughout the lithotripsy session, rather than waiting until its completion, may reduce the fragment burden available for distal migration. Positioning the patient in the left lateral position, rather than supine, may also slow gravity-dependent passage of fragments and facilitate their retrieval. Close postprocedural monitoring remains essential, particularly in high-risk patients in whom further intervention may not be feasible. It should be noted that these recommendations are currently hypothesis generating rather than evidence based, as no prospective data exist demonstrating that staged fragment retrieval or patient positioning reduces distal complications.

## DISCLOSURES

Author contributions: MN Vankina drafted the manuscript and performed literature review. J. Sreenarasimhaiah supervised, edited, and approved the final manuscript. All authors approved the final version. MN Vankina is the article guarantor.

Financial disclosure: None to report.

Previous presentation: This case was presented as a poster at the 2025 American College of Gastroenterology Annual Scientific Meeting; October 28, 2025; Phoenix, AZ.

Informed consent could not be obtained for this case report. All identifying information has been removed.

Artificial intelligence disclosure: ChatGPT was used for minor language editing and formatting. All content was reviewed and approved by the authors, who take full responsibility for the manuscript.

## ABBREVIATIONS:

CT: computer tomography
